# 90-Gene Expression Profiling for Tissue Origin Diagnosis of Cancer of Unknown Primary

**DOI:** 10.3389/fonc.2021.722808

**Published:** 2021-10-07

**Authors:** Yi Zhang, Lei Xia, Dawei Ma, Jing Wu, Xinyu Xu, Youtao Xu

**Affiliations:** ^1^ Department of Pathology, Jiangsu Cancer Hospital, Jiangsu Institute of Cancer Research, The Affiliated Cancer Hospital of Nanjing Medical University, Nanjing, China; ^2^ Department of Radiation Oncology, Jiangsu Cancer Hospital, Jiangsu Institute of Cancer Research, The Affiliated Cancer Hospital of Nanjing Medical University, Nanjing, China; ^3^ Department of Thoracic Surgery, Jiangsu Cancer Hospital, Jiangsu Institute of Cancer Research, The Affiliated Cancer Hospital of Nanjing Medical University, Nanjing, China

**Keywords:** cancer of unknown primary, exome panel sequencing, expression characteristics, FFPE, RT-PCR

## Abstract

Cancer of unknown primary (CUP), in which metastatic diseases exist without an identifiable primary location, accounts for about 3–5% of all cancer diagnoses. Successful diagnosis and treatment of such patients are difficult. This study aimed to assess the expression characteristics of 90 genes as a method of identifying the primary site from CUP samples. We validated a 90-gene expression assay and explored its potential diagnostic utility in 44 patients at Jiangsu Cancer Hospital. For each specimen, the expression of 90 tumor-specific genes in malignant tumors was analyzed, and similarity scores were obtained. The types of malignant tumors predicted were compared with the reference diagnosis to calculate the accuracy. In addition, we verified the consistency of the expression profiles of the 90 genes in CUP secondary malignancies and metastatic malignancies in The Cancer Genome Atlas. We also reported a detailed description of the next-generation coding sequences for CUP patients. For each clinical medical specimen collected, the type of malignant tumor predicted and analyzed by the 90-gene expression assay was compared with its reference diagnosis, and the overall accuracy was 95.4%. In addition, the 90-gene expression profile generally accurately classified CUP into the cluster of its primary tumor. Sequencing of the exome transcriptome containing 556 high-frequency gene mutation oncogenes was not significantly related to the 90 genes analysis. Our results demonstrate that the expression characteristics of these 90 genes can be used as a powerful tool to accurately identify the primary sites of CUP. In the future, the inclusion of the 90-gene expression assay in pathological diagnosis will help oncologists use precise treatments, thereby improving the care and outcomes of CUP patients.

## Introduction

Cancer of unknown primary (CUP) is a term applied to a group of heterogeneous metastatic malignancies whose primary location cannot be detected when the tumor migrates (1). In such cases, general investigations are unable to clarify the primary location at the time of diagnosis. CUP is the seventh to eighth most common cancer and is the fourth most common cause of cancer-related deaths (2). Although several advances have been made with specialized tools for cancer diagnosis, and the incidence of CUP has steadily reduced from 5% to approximately 2% of all newly diagnosed invasive cancers, the prognosis of CUP patients is still poor. Therefore, a precise diagnosis is essential for CUP patients to allow site-specific treatment and to improve their outcomes (3).

In those diagnosed with CUP, only about 20% share clinicopathological characteristics with particular known metastatic cancers. Median survival in this group may be as long as 24 months with treatment directed at the likely primary site, usually under the supervision of the relevant site-specific treatment (4). The remaining 80% response to systemic therapy is often limited, with a median survival of 6–9 months (5). In addition, the lack of primary tumor definition, prevent most patients to be treated in clinical practice with a novel, very effective treatment such as immunotherapy or molecular targeted therapies for which currently registered indications are mostly disease-oriented. Thus, an accurate diagnosis is urgently needed to identify the most probably site-of-origin or an approach based on personalized medicine. It is useful to assist in the selection of the best treatment options and potentially improve CUPs prognosis and survival.

Generally, the diagnosis of CUP is based on the European Society of Medical Oncology guidelines (6). Clinical manifestations, tumor markers, and imaging diagnostic analyses are used to clarify the origin of metastatic cancer. In clinical practice, histopathologic and immunohistochemistry (IHC) analyses are still particularly important for identifying the anatomical origin of CUP patients. However, these traditional approaches become difficult when the hypothetical primary malignant tumor grows too large to be identified before it migrates (7).

As an alternative, molecular structure analysis of malignant tumors is a promising technology that can improve the diagnosis of the origin in CUP patients (8). Currently, there are several available testing methods that use reverse transcription-polymerase chain reaction (RT-PCR) or genetic microarray technology (9). In a previous study, a microarray-based 1550-gene expression profile was used to distinguish the primary site in 13 specimens known to originate from brain metastases, and excellent results were obtained with an accuracy of 92.3% (10). Another study revealed the expression characteristics of 154 genes, which could accurately classify 21 common types of malignant tumors (11). It is hoped that this gene panel will become an effective tool for identifying the origin of malignant tumors (12). The RT-PCR technology is more convenient and accurate than the microarray technology. It can also be used for formalin-fixed paraffin-embedded (FFPE) specimen collection and is widely used in clinical medicine (13).

In this study, we investigated the accuracy of a 90-gene expression assay for classifying 21 types of malignant tumors, in comparison with the actual disease diagnosis. In a multisite study, the performance of the 90-gene expression assay was illustrated in 609 tumor samples of known primary origin, with an accuracy of 89.8%. More specifically, the classification accuracy reached 90.4% in primary tumors and 89.2% in metastatic tumors (14).In addition, a full exome analysis was carried out to find the mutation spectrum that is likely to be beneficial for revealing the vulnerabilities of CUP patients.

## Materials and Methods

### Patient and FFPE Specimen Collection

CUP samples of 44 patients archived from May 2018 to December 2020 were used. All hematoxylin and eosin-stained slides were assessed by two pathologists to ensure consistency with the reference diagnosis and certify the percentage of malignant cells. The inclusion criteria were as follows: i) availability of FFPE tumor tissue samples, ii) disease diagnosis included in the 21 types of tumors for the 90-gene panel, and iii) at least 60% tumor cell content based on hematoxylin and eosin-stained slides. Clinical data, physical examination information, and results of imaging, light microscopy, and IHC tests were obtained from medical records.

### Details of the 90 Tumor-Specific Genes

Initially, the tumor-specific genes were identified on basis of a pan-cancer transcriptome database comprising 5434 samples representing 21 tumor types (14). Next, the Top-10 most predictive genes for each of the 21 tumor types were screened by using the Support Vector Machine Recursive Feature Elimination (SVM-RFE) algorithm. A list of 90 genes corresponding to 21 tumor types was identified after removing redundant genes (**Additional Table 1**). Finally, an SVM linear model was trained using the whole pan-cancer transcriptome database to establish a multiclass classification algorithm termed “90-gene classifier”. The details of the 90 specific genes were list in the **Additional Table 2**.

Intuitively, the similarity scores for each of the 21 tumor types were calculated by the 90-gene classifier, which reflect how much the gene expression pattern of the test specimen is similar to the global gene expression pattern with known tumor type. The similarity scores were probability-based, with a reported range from 0 to 100, and all 21 similarity scores sum to 100. The tumor type with the highest similarity score was considered as the predicted tumor type by the 90-gene classifier.

### Sample Preparation and RNA Isolation

An FFPE Total RNA Isolation Test Kit (Canhelp Genomics, Hangzhou, China) was used to isolate total RNA from FFPE samples, as described previously. Briefly, tumor sections were placed in a small 1.5 mL centrifuge tube, deparaffinized with xylene at 50°C for 3 minutes, and then washed twice with 100% alcohol. The samples were incubated in a trypsin K aqueous solution at 56°C for 15 minutes and subsequently at 80°C for another 15 minutes. DNase was then used to digest and absorb the protein. We used 40 μL of RNase-free water to obtain the total RNA. The concentration of total RNA was measured with a 260 nm photometer, and the purity was measured by the A260/A280 ratio. The RT-PCR (Applied Biosystems 7500) analysis was only carried out on RNA samples with A260/A280 ratios between 1.2 and 2.4.

### Expression Profiling of 90 Tumor-Specific Genes

For each sample, cDNA was generated from total isolated RNA using a High-Capacity cDNA Reverse Transcription Kit with RNase Inhibitor (Applied Biosystems, Foster City, CA, United States). RT-PCR was used to analyze the expression profiles of 90 specific genes in malignant tumors on a 96-well plate.

### Downloading Public Data and Analyses

RNA expression profiles (workflow type: HT Seq-Counts) and the patients’ clinical information were downloaded from The Cancer Genome Atlas website using the “TGCA biolinks” R package (Version 2.14.1). In order to acquire the relative expression of each mRNA, we conducted normalize each counts *via* a standard pipeline of “DESeq2” R package (Version 4.1). “Combat” function of “sva” R package (Version 3.0) was used to removed batch effects between our panel profiling and TCGA RNA-seq.

### Library Preparation and Sequencing

For targeted therapeutic transcriptome sequencing, genomic DNA from FFPE sections or biopsy samples and the whole blood control samples were extracted with QIAamp DNA FFPE Tissue Kit and DNeasy Blood and tissue kit (Qiagen), respectively, while cfDNAs from whole blood which were collected with Streck Cell-Free DNA BCT were extracted with QIAamp Circulating Nucleic Acid Kit (Qiagen) and then quantified with Qubit 3.0 using the dsDNA HS Assay Kit (Thermo-Fisher Scientific). Library preparations were performed using the KAPA HyperPlus Prep Kit (KAPA Biosystems). For the targeted panel, customized xGen lockdown probes (Integrated DNA Technologies) targeting 556 cancer-relevant genes were used for hybridization enrichment. The capture reaction was performed with Dynabeads M-270 (Life Technologies) and the xGen Lockdown Hybridization and Wash Kit (Integrated DNA Technologies), according to the manufacturers’ protocols. Captured libraries were subjected to on-beads PCR amplification with Illumina p5 (5’ AAT GAT ACG GCG ACC ACC GA 3’) and p7 primers (5’ CAA GCA GAA GAC GGC ATA CGA GAT 3’) using the KAPA HiFi HotStart ReadyMix (KAPA Biosystems), followed by purification using Agencourt AMPure XP beads. Libraries were quantified by qPCR using the KAPA Library Quantification Kit (KAPA Biosystems). The library fragment size was determined using the Bioanalyzer 4200 (Agilent Technologies). The target-enriched library was then sequenced on a NovaSeq 6000 system (Illumina) according to the manufacturer’s instructions, with an average coverage depth of 2000× for tumors and 8000× for cfDNA (500× in normal blood controls) using a panel. The average mask size of the panel was 2.8 Mb.

### Statistical Analysis

Gene expression data analysis was performed using R software and packages from the Bioconductor project. The gene expression mode of each specimen was compared with 21 specific types of malignant tumors based on the expression characteristics of 90 genes (**Additional Tables 1, 2**). For each of the 21 types of malignant tumors, similarity scores were calculated, which indicated the similarities of the intermediate gene expression patterns between the template and the specific malignant tumor type. The similarity score ranged from 0 (very low similarity) to 100 (very high similarity). The type of malignant tumor with the highest similarity score was considered to indicate the origin. For each specimen, the primary location of the malignant tumor was predicted and analyzed in comparison with the clinical reference diagnosis.

## Results

### Patients and Samples

**Figure 1:**
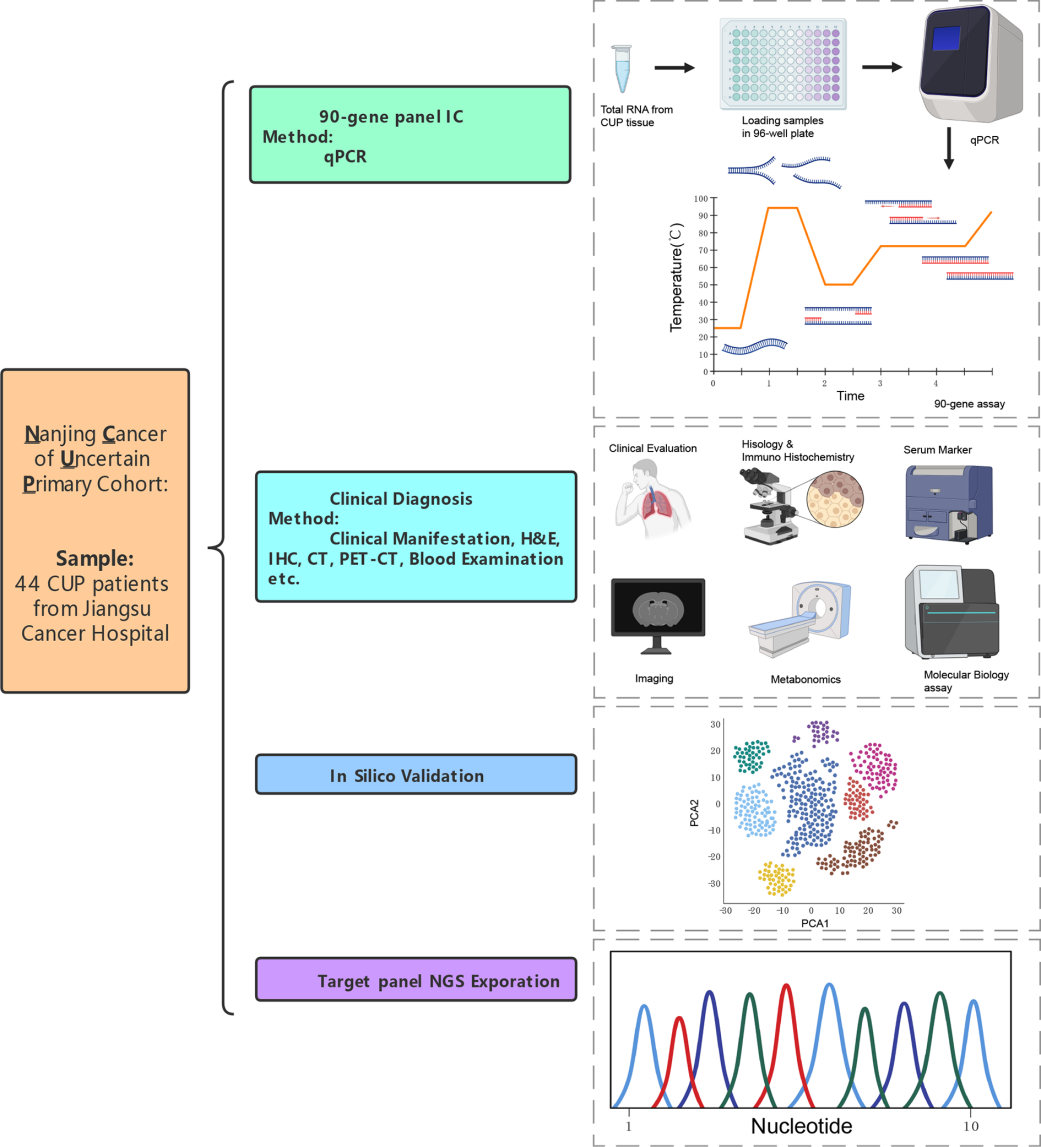
An overview of research design that verification of 90-gene expression signature to identify the origin of CUP.

**Figure 2:**
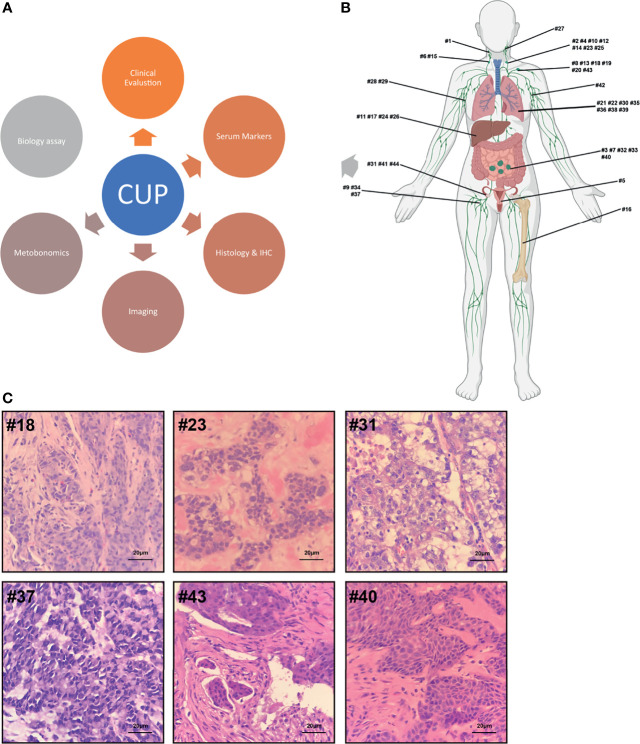
**(A)** Multistep origin of CUP diagnostic workflow. **(B)** Metastases distribution: The 44 metastases were retrieved from surgery and puncture biopsy specimens from 44 CUP patients. **(C)** Histology: Most metastatic lesions are composed of poorly differentiated.

The flow diagram for the study participants is shown in **Figure 1**. From May 2018 to December 2020, 44 patients were recruited from Jiangsu Oncology Hospital for this study. As shown in **Table 1**, the study included 23 men and 21 women with a mean age of 59 years (range 32–89 years). The 44 specimens were classified into 12 types based on the location of malignant tumor invasion, including the cervical lymph nodes, axillary lymph nodes, groin lymph nodes, head and neck, lungs, liver, female genitalia, omentum, abdominal cavity, retroperitoneum, bone, and sternum. The most common erosion sites for the first, second, and third malignant tumors were the cervical lymph nodes (20.4%), head and neck (18.2%), and lungs (13.6%), respectively. Among the 44 specimens collected, 15 (34%) were well-differentiated tumors and 29 (66%) were poorly differentiated. In accordance with the European Society of Medical Oncology manual, a comprehensive CUP multi-process exercise was performed to eliminate the presence of primary malignant tumors (**Figure 2**).

**Table 1:** Patients and clinical characteristics.

Characteristic	No. of specimens	Percentage (%)
**Gender**		
Male	23	53
Female	21	47
**Age at diagnosis**		
Mean	59
Range	32-89
**Diagnostic Method**		
Biopsy	24	54.5
Surgery	20	45.5
**Invasion site**		
Cervical Lymph nodes	9	20.4
Axillary Lymph nodes	3	6.8
Inguinal Lymph nodes	3	6.8
Neck	8	18.2
Lung	6	13.6
Liver	4	9
Female reproductive	4	9
Omentum	2	4.5
Abdomen	2	4.5
Peritoneum	1	2.2
Bone	1	2.2
Chest wall	1	2.2
**Histology**		
Well-differentiated	15	34
Poorly-differentiated	29	66

### The Characteristics of the Expression Characteristics of 90 Genes in CUP

**Figure 3:**
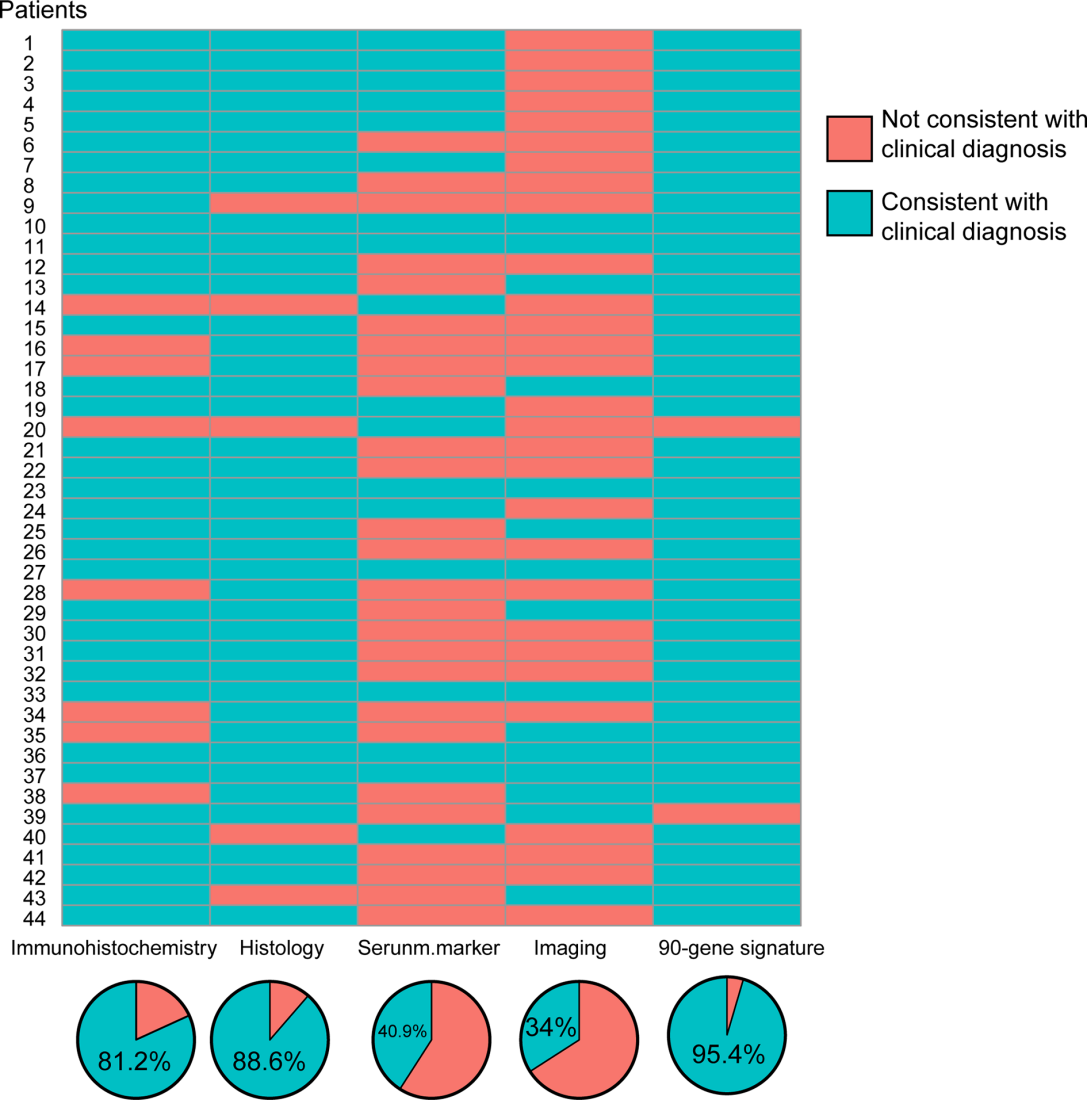
Consistency and coincidence rate of CUP origin multidisciplinary diagnosis results with 90-gene expression signature.

Total RNA was isolated from tissue sections of the 44 samples. The concentration range was 2.59–653 ng/µL, with an average of 106 ng/µL. The A260/A280 ratio ranged from 1.2 to 2.4. In **Figure 3**, the red grid represents the non-compliance with the reference diagnosis, and the blue grid represents the compliance with the clinically predicted diagnosis. After analyzing the clinical data of these forty-four patients, it was found that the diagnosis coincidence rate was 81.2% based on immunohistochemistry analysis; the diagnosis coincidence rate based on morphological analysis was 88.6%; the diagnosis coincidence rate based on serological examination was 40.9% and the coincidence rate based on imaging diagnosis alone was 34%. Of the 44 samples, 95.4% (42/44) showed agreement between the prediction of the 90-gene expression assay and the reference diagnosis.

The molecular structure classifications were inconsistent in two of the specimens, as shown in **Table 2**. In one case, a malignant tumor of the cervical lymph nodes was predicted to be liver adenocarcinoma, but the IHC marker for liver adenocarcinoma was negative. The reason for the case is the limitation of IHC and the tumor heterogeneity that affects antibody expression. The other inconsistency was from a patient whose malignant tumor was diagnosed by pathophysiologic examination as a poorly differentiated cancer that migrated to the lung, but was assessed as sarcoma by the 90-gene expression analysis. The excuse can be due to the abundant necrosis decreased the amount of entity of tumor, and meanwhile the scant components of tumor affected the accuracy of 90-gene analysis. This phenomenon of necrosis is the reflected response to radiotherapy and chemotherapy treatment and always be the consequence of insufficient blood supply.

**Table 2:** Investigation of cases with discordant 90-gene expression.

ID	Reference diagnosis	History	Immunohistochemical staining	90-gene expression results	Similarity score
**#20**	Poorly differentiated cancer	A 48-year-old man with tumor in cervical lymph nodes	AE1/AE3++; CK5/6-; P40-; CK7+; TTF-1-; CK20-; Hep-1-; Arg-1-; PAX-8-; GATA-3-; CDX-2-.	Hepatobiliary tumors	54.3
**#39**	Lung epithelial tumor	A 49-year-old man with a tumor in the lung	AE1/AE3+; Syn-; CgA-; NapsinA+; PAX-8-; GATA-3-; CDX-2-.	sarcoma	48.8

### *In-Silico* Validation of the 90 Gene Expression Signature in CUP

**Figure 4:**
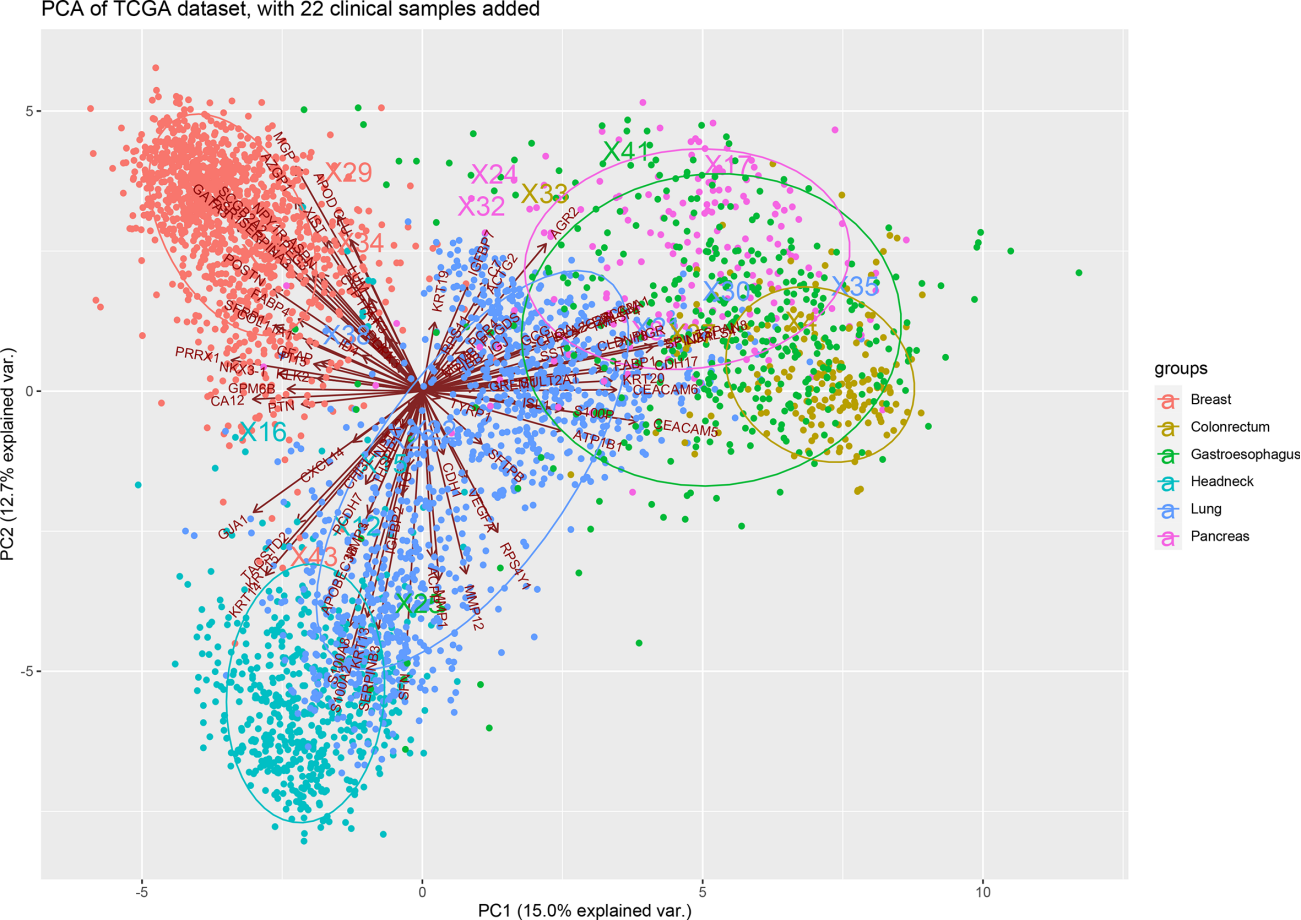
Principal component analysis of 90-gene expression between CUP and primary tumor from TCGA database.

To further validate the performance of the 90-gene expression signature in CUP, we evaluated the consistency of the 90-gene expression profile between the CUP primary tumor and metastatic tumor. We downloaded the transcriptome data of lung adenocarcinoma, lung squamous cell carcinoma, gastric adenocarcinoma, head and neck squamous cell carcinoma, breast cancer, and pancreatic cancer from The Cancer Genome Atlas database and extracted the 90-gene expression signatures. Satisfactorily, the 90-gene expression profile generally accurately classifies CUP into the cluster of its primary tumor (**Figure 4**), suggesting that the 90-gene expression signal can cover the transcriptome characteristics of the patient’s primary tumor.

### Exploration of Exome Panel Sequencing in CUP Patients

**Figure 5:**
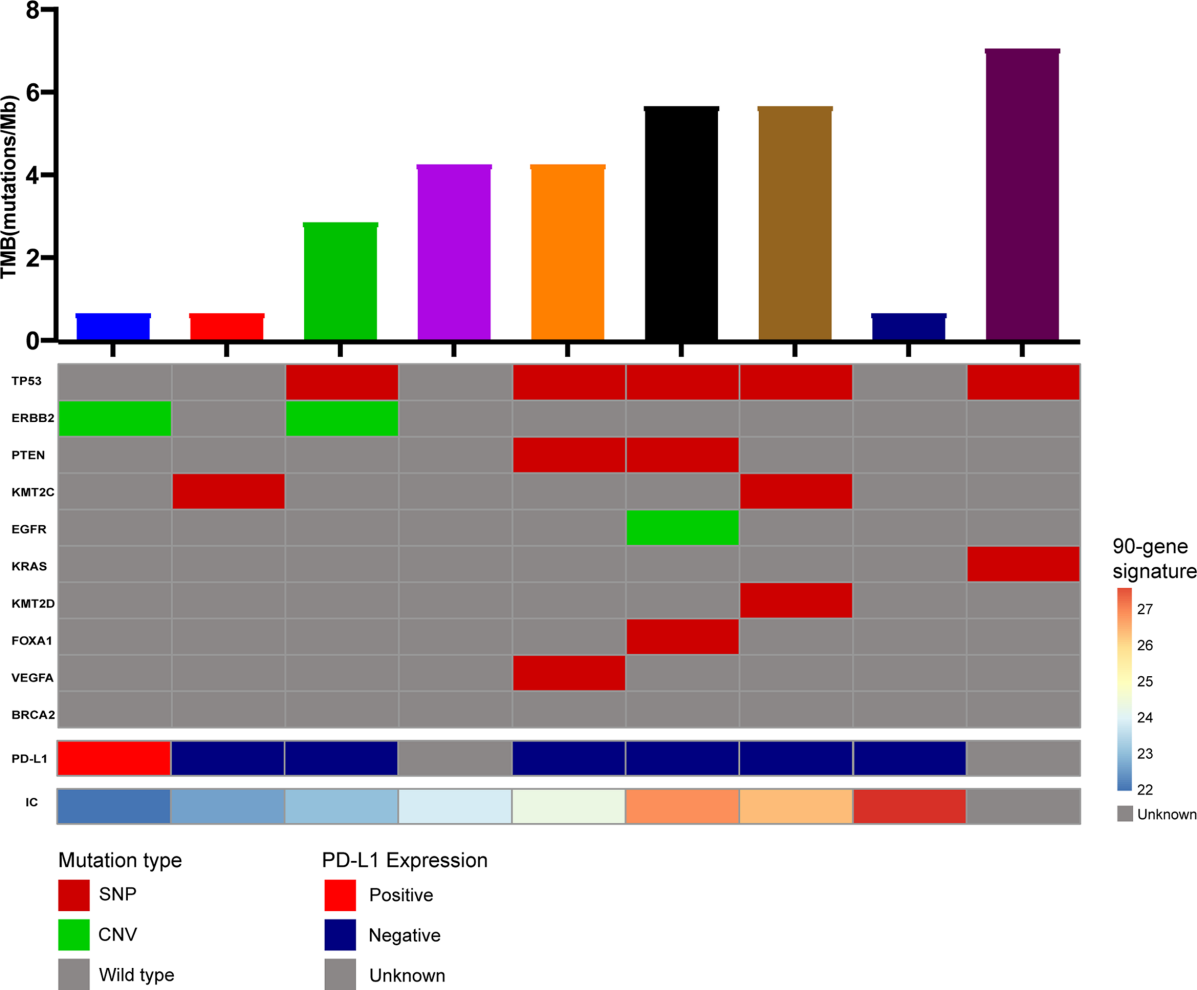
NGS panel sequencing of metastatic tissues from 9 CUP patients.

Moreover, we conducted exome panel sequencing in nine patients with cervical lymph node metastatic tumors. We performed a high-throughput exome sequencing technology containing 556 cancer-related genes. It was a pan-solid tumor-associated big panel that includes comprehensive mutational information to guide targeted therapy and immunotherapy. Moreover, gene associated with sensitivity or tolerance to chemoradiotherapy was also included to help optimize treatment options (**Figure 5**). Unfortunately, the single nucleotide polymorphisms and copy number variations of the 556 genes did not correlate significantly with the expression tag of the 90 genes, nor with tumor mutation burden or *PD-L1* expression. This also reflects the irreplaceable expression profile of the 90-gene expression signature.

## Discussion

In the present study, the expression characteristics of 90 genes were validated in CUP patients, and the overall agreement with the reference diagnosis was 95.4%. Although the expression characteristics of 90 genes mainly showed accurate aspect ratios for classifying CUP malignant tumors, we noticed that two medical records were incorrectly classified. In one case, a negative result was obtained from an IHC analysis that used markers for malignant tumors, indicating that the pathological diagnosis was limited.

When addressing the potential diagnosis of CUP, clinical practice suggests a complete diagnosis based on the European Society of Medical Oncology guidelines (15). The diagnostic test is based on the precise location of the migration foci, the patient’s clinical symptoms and sex, magnetic resonance imaging, endoscopy, positron emission tomography, or evaluation of special serum protein tumor markers. The final step is IHC testing, which is still the most important diagnostic tool for identifying the origin (16). In 69% of cases, histological or IHC analysis clarified the primary location of a series of poorly differentiated tumors (17). Many molecular structure tests based on gene expression profiles have shown useful value in identifying the type of primary malignant tumors in patients with unknown or uncertain diagnoses (18).

A previous meta-analysis showed that IHC provided appropriate origin identification in only 65.6% of metastatic cancers. Recent studies have also shown that molecular structure profiling is better than IHC classification, especially in cases of poorly differentiated malignant tumors (19). The second incorrectly classified case was a metastatic malignant tumor with a wide area of necrosis and was classified as sarcoma based on gene expression. A large amount of necrosis reduced the total number of solid lines of malignant tumors, and the lack of malignant tumor components affected the accuracy of the 90-gene expression assay. This type of necrosis reflects radiotherapy and chemotherapy and is usually the result of insufficient blood supply (20). Therefore, 90-gene expression assay can be used to completely classify these undiagnosed malignancies.

In the molecular era, gene expression profiling and next-generation sequencing have been proposed to identify the site of origin and to replace the standard pathological examinations based on histologic examination and IHC (21). Next-generation sequencing panels are still the most common way to identify the primary location of CUP and establish targeted drug therapy (22). However, our study showed that the origin of CUP cannot be confirmed based on the results of a common next-generation sequencing panel with transcriptome sequencing (including gene mutation, copy number variation, microsatellite instability, and tumor mutational burden). This may be due to the heterogeneity of intermediate genes between primary and migrating malignancies (23). For example, case #44 in our study involved liver metastasis from a mutation in the *KRAS p.G12V* gene, which is a common mutation in pancreatic tumors. Comprehensive clinical symptoms, pathologic examination, imaging diagnosis, and serological examination all indicated that the primary malignant tumor originated from the colon. In addition, the composition of *BRAF* and *MEK* inhibitors is reasonable for melanoma patients with *BRAF V600* mutations but has limited efficacy for patients with rectal cancer (24).

Although research on predicting the origin of CUP through transcriptome data is gaining popularity, there is a widespread lack of clinical research certification and clinical medical applications, especially in China (25). Since RNA sequencing requires a lot of resources for specimen collection, the results are unstable, and a large financial burden is placed on the patient. A simple, cheap, and stable PCR method must be applied to assess the expression levels of 90 genes in patients. In a previous study, a 92-gene control panel developed by Ma et al. was able to distinguish the origin of CUP with an overall sensitivity of 87% (26). In another study, a 10-gene control panel could distinguish between six types of multiple malignancies. These reflect the feasibility of PCR to assess the origin of CUP (27). Moran et al. developed and designed a DNA methylation profile for CUP patients with an overall accuracy of 90% (28). This inspired us to further improve the accuracy of CUP origin identification from an epigenetic perspective in the future.

In our study, the 90-gene expression profile was similar for metastatic malignant tumors and primary malignant tumors. This shows that secondary malignant tumors and metastatic malignant tumors have molecular structures similar to those of malignant progression. This provides an identification method for CUP and the possible vulnerabilities of CUP patients, including ASPN (29), GATA3 (30), and VEGF-A (31). Meanwhile, some scientific research groups have reported many genetic changes in CUP malignant tumor origins or liquid biopsies (32). In addition, studies investigating the presence of driver mutations and molecular aberrations in CUP provide conflicting evidence on whether these changes are “potentially druggable” (33).

In conclusion, this 90-gene expression assay can identify the source of malignant tumors with good accuracy, and the expression data can cover the transcriptome characteristics of the patient’s primary malignant tumor. In addition, we carried out exome transcriptome sequencing, including 556 high-frequency gene mutation oncogenes, to explore the gene expression of CUP patients.

## Data Availability Statement

The datasets presented in this study can be found in online repositories. The names of the repository/repositories and accession number(s) can be found below: National Genomics Data Center, HRA001047.

## Ethics Statement

The studies involving human participants were reviewed and approved by Nanjing Medical University. The patients/participants provided their written informed consent to participate in this study. Written informed consent was obtained from the individual(s) for the publication of any potentially identifiable images or data included in this article.

## Author Contributions

YZ, LX, and DM participated in the study design and data analysis and draft the manuscript. JW and YX carried out the immunoassays and help to draft the manuscript. XX conceived the study, participated in its design and coordination, and help to draft the manuscript. All authors contributed to the article and approved the submitted version.

## Funding

This work was partially supported by grants from Jiangsu Cancer Hospital (numbers ZM202006), the National Natural Science Foundation of China (number 81702692), the Jiangsu Province “Six Talent Peaks Project” (WSW-019).

## Conflict of Interest

The authors declare that the research was conducted in the absence of any commercial or financial relationships that could be construed as a potential conflict of interest.

## Publisher’s Note

All claims expressed in this article are solely those of the authors and do not necessarily represent those of their affiliated organizations, or those of the publisher, the editors and the reviewers. Any product that may be evaluated in this article, or claim that may be made by its manufacturer, is not guaranteed or endorsed by the publisher.

## Abbreviations

CUP, cancer of unknown primary; FFPE, formalin-fixed paraffin-embedded; IHC, immunohistochemistry; RT-PCR, reverse transcription-polymerase chain reaction; ASPN, Asporin; GATA3, GATA binding protein 3; VEGF-A, vascular endothelial growth factor A; AE1/AE3, pancytokeratin; CK5/6, Cytokeratin 5/6; P40, polypeptide 40; CK7, Cytokeratin7; TTF-1, Thyroid transcription factor‐1; CK20, Cytokeratin 20; Hep-1, hepatocyte paraffin 1; Arg-1, Arginase-1; PAX-8, The paired box transcription factor; CDX-2, Caudal-related homeobox transcription factor 2; Syn, synaptophysin; CgA, chromogranin A; NapsinA, cytoplasmic aspartic protease.
